# ‘*Candidatus* Rickettsia andeanae’ and Probable Exclusion of *Rickettsia parkeri* in Ticks from Dogs in a Natural Area of the Pampa Biome in Brazil

**DOI:** 10.3390/pathogens12030446

**Published:** 2023-03-12

**Authors:** Felipe S. Krawczak, Lina C. Binder, Fábio Gregori, Thiago F. Martins, Gracielle T. Pádua, Jonas Sponchiado, Geruza L. Melo, Gina Polo, Marcelo B. Labruna

**Affiliations:** 1 Setor de Medicina Veterinária Preventiva, Departamento de Medicina Veterinária, Escola de Veterinária e Zootecnia, Universidade Federal de Goiás, Rod. Goiânia—Nova Veneza, km 8, Campus Samambaia, Goiânia 74690-900, GO, Brazil; 2 Departamento de Medicina Veterinária Preventiva e Saúde Animal, Faculdade de Medicina Veterinária e Zootecnia, Universidade de São Paulo, Av. Prof. Orlando Marques de Paiva, 87, Cidade Universitária, São Paulo 05508-270, SP, Brazil; 3 Instituto Federal de Educação, Ciência e Tecnologia Farroupilha, Campus Alegrete, Alegrete 97541-000, RS, Brazil; 4 Facultad de Medicina Veterinaria y Zootecnia, Fundación Universitaria San Martin, Bogotá 110110, DC, Colombia

**Keywords:** *Amblyomma tigrinum*, ‘*Candidatus* R. andeanae’, dogs, *Amblyomma aureolatum*

## Abstract

Spotted fever illness caused by the tick-borne pathogen *Rickettsia parkeri* has emerged in the Pampa biome in southern Brazil, where the tick *Amblyomma tigrinum* is implicated as the main vector. Because domestic dogs are commonly parasitized by *A. tigrinum,* this canid is also a suitable sentinel for *R. parkeri*-associated spotted fever. Herein, we investigate rickettsial infection in ticks, domestic dogs and small mammals in a natural reserve of the Pampa biome in southern Brazil. The ticks *A. tigrinum, Amblyomma aureolatum* and *Rhipicephalus sanguineus* were collected from dogs. Molecular analyses of ticks did not detect *R. parkeri*; however, at least 34% (21/61) of the *A. tigrinum* ticks were infected by the non-pathogenic agent ‘*Candidatus* Rickettsia andeanae’. Serological analyses revealed that only 14% and 3% of 36 dogs and 34 small mammals, respectively, were exposed to rickettsial antigens. These results indicate that the study area is not endemic for *R. parkeri* rickettsiosis. We tabulated 10 studies that reported rickettsial infection in *A. tigrinum* populations from South America. There was a strong negative correlation between the infection rates by *R. parkeri* and ‘*Candidatus* R. andeanae’ in *A. tigrinum* populations. We propose that high infection rates by ‘*Candidatus* R. andeanae’ might promote the exclusion of *R. parkeri* from *A. tigrinum* populations. The mechanisms for such exclusion are yet to be elucidated.

## 1. Introduction

South America has experienced the emergence and re-emergence of tick-borne spotted fever illness affecting humans and domestic dogs. In Brazil, the reported cases of tick-borne spotted fever are associated with different species of *Rickettsia*, such as *Rickettsia rickettsii*, *Rickettsia parkeri* sensu stricto (s.s.) and the *Rickettsia parkeri* strain, Atlantic rainforest [1,2,3,4,5].

Rio Grande do Sul, the southernmost state in Brazil, is the only part of the country where the Pampa biome can be found. The Pampa biome has a temperate climate with four well-defined seasons and rainfall distributed throughout the year, and is located within the Temperate Zone [6]. At least 14 confirmed cases of tick-borne spotted fever were reported from 2007 to September 2022 in Rio Grande do Sul, where the possible etiological agent involved is *R. parkeri* [7], either *R. parkeri* s.s. [4] or *R. parkeri* strain Atlantic rainforest [8]. In the Pampa biome, the species *Amblyomma triste* and *Amblyomma tigrinum* are commonly found on domestic and wild Carnivora, and these ixodid species are considered the main vectors of *R. parkeri* s.s. [4,9,10,11]. In Argentina, Uruguay and the Brazilian Pampa Biome, *R. parkeri* s.s. has been described as the main causative agent of spotted fever rickettsiosis [4,10,11,12]. 

The bacterium ‘*Candidatus* Rickettsia andeanae’, although classified as a spotted fever group agent, is not known to cause human illness [13]. It has been proposed that the infection of ‘*Candidatus* R. andeanae’ in a tick population might interfere with *R. parkeri* development in ticks [13]. 

Recent studies have indicated an increase in the known distribution area of *R. parkeri* s.s. in the Pampa biome and suggest that this pathogen may be much more common in this region than previously reported [14,15,16,17,18]. Epidemiological studies have indicated the domestic dog as being a highly suitable indicator of the *R. parkeri*-associated spotted fever in southern Brazil [8,19]. Therefore, the aim of this study was to investigate rickettsial infection in ticks, small mammals and domestic dogs in a natural reserve of the Pampa biome in Rio Grande do Sul, Brazil. 

## 2. Materials and Methods

### 2.1. Study Area

This study was performed in Espinilho State Park “Parque Estadual do Espinilho” (PEE) (30°12′25″ S; 57°33′18″ W) and in the rural area surrounding the Park. The Park has an area of 1617.14 ha and is located in the municipality of Barra do Quaraí, the westernmost city in the southern region of Brazil. The PEE is a natural Reserve of the Pampa biome, which in Brazil is restricted to the state of Rio Grande do Sul. The climate of the PEE region is Cfa type according Koppen-Geiger, characterized by a humid temperate climate with hot summers, and an average annual temperature of 23.4 °C and annual precipitation of 1300 mm. The municipality of Barra do Quaraí had an estimated population of 4238 inhabitants in 2021 and a territorial area of 105,593.7 ha. (https://www.ibge.gov.br/cidades-e-estados/rs/barra-do-quarai.html, accessed on 01 July 2022).

According to the PEE Management Plan, it is estimated that 32 species of mammals inhabit the region [20], 10 of which are on the list of endangered species of wild fauna of the State of Rio Grande do Sul, such as *Alouatta caraya* (black howler monkey)*, Leopardus munoai* (pampas cat)*, Lepardus geoffroyi* (Geoffroy’s cat)*, Herpailurus yaguarondi* (jaguarundi)*, Chrysocyon brachyurus* (maned wolf)*, Nasua nasua* (South American coati)*, Ozotoceros bezoarticus* (pampas deer) and *Wilfredomys oenax* (greater Wilfred’s mouse).

Three field campaigns were performed, the first in June 2013, the second in October 2013, and the third in January 2014. In each one, we sampled domestic dogs, small mammals and ticks. Field campaigns and animal procedures described here have been authorized by the Chico Mendes Institute for Biodiversity (ICMBio Permit No. 38502-1), the Institutional Animal Care and Use Committee (IACUC) of the School of Veterinary Medicine and Animal Science of the University of São Paulo (protocol 2908/2013), and State Secretary for the Environment of the Rio Grande do Sul (protocol 05/2013 registration No. 428).

### 2.2. Domestic Dogs

During the three field campaigns in PEE and in the surrounding rural area, 36 individual dogs had blood serum samples collected. In the first field campaign, 33 of these dogs were examined for tick infestations. During the second and third field campaigns, 17 and 20 of the first campaign-dogs, respectively, were re-examined for tick infestations, resulting in a total of 70 canine examinations during the study. Individual canine examinations for ticks were performed as described next [21]. Briefly, it consisted of a 3–4 min examination period of the entire canine body, from which the collected ticks were put in plastic tubes and transported alive to the laboratory.

For calculation of the number of sampled dogs to be tested in serological analysis, we adopted a 3.16:1 ratio (number of humans/number of dogs) for rural area, following Soto et al. [22]. The sample size is considered 10% of the expected prevalence, based on a previous study that evaluated canine seroreactivity to spotted fever group (SFG) rickettsiae [23], with 95% accuracy. Thus, the formula of Arya et al. [24] was adopted, as follows: 

*n* = [(*z*²)*P*(1 − *P*)]/d², where *n* = sample size; *z* = z statistic for the level of confidence; *P* = expected prevalence; and *d* = allowable error. This formula allowed for at least 28 dogs to be sampled in the present study.

### 2.3. Small Mammals

**Figure 1:**
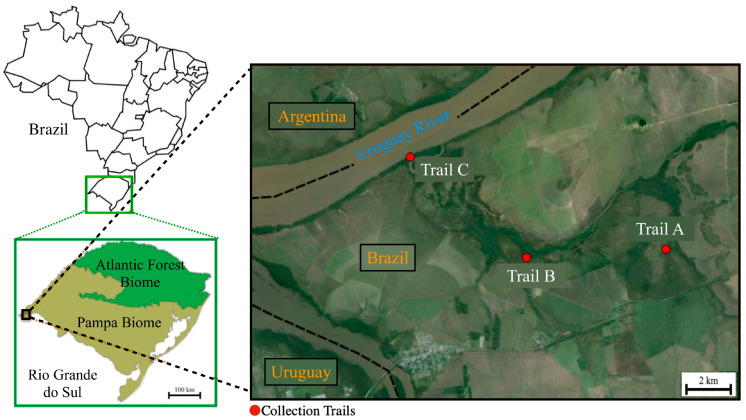
Locations of trails A, B and C within the Espinilho State Park in the state of Rio Grande do Sul, southern Brazil, where animals and ticks were sampled in the present study.

Field captures of wild small mammals were attempted in three trails (A, B and C) within the PEE (Figure 1). For this purpose, we used a total of 75 Shermman (25 in each trail) and five Tomahawk live-traps (two in trail A, two in trail B, and one in trail C) baited with banana, bacon, peanut butter, apple, and ham. 

In each of the three campaigns, the traps were used during four consecutive nights. Concomitantly, three stations of pitfall traps were set up by using five buckets of 42.5 cm diameter and 60 cm height connected to a plastic fence (of least 30 m long and 50 cm high) in each station [25]. The total sampling effort was 960 trap-nights for live traps and 180 trap-nights for pitfall traps. Captured mammals were taxonomically identified following previous studies [26,27], submitted to anesthesia by using ketamine and xylazine, and then examined for tick infestations. Each animal was bled through intracardiac or tail vein route, and the blood sample was centrifuged for obtaining sera to be tested by serological analysis. A numbered earring (fish and small animal tag size 1; National Band and Tag, Newport, KY, USA) was applied to each animal. After returning from anesthesia, the small mammals were released at the same trapping site.

### 2.4. Host-Seeking Ticks

In each of the three campaigns, collection of host-seeking ticks from ground vegetation consisted of passing a cotton flannel (75 × 100 cm) by one investigator through a 50–100 m trail, as described in [21,28]. In parallel, we used the visual search method, as described in [29]. Collected ticks were transported alive to the laboratory.

### 2.5. Tick Identification and Rickettsial Infection in Ticks

Taxonomic identification of ticks relied on standard morphological keys [30,31,32]. Adult ticks that arrived alive at the laboratory were stored in a −80 °C freezer until they were tested for isolation of rickettsiae in Vero cell culture, through the technique of shell vial, as previously described in [33]. 

Other frozen or alcohol-preserved adults and the body remnants of ticks processed by the shell vial technique had their DNA extracted through the guanidine isothiocyanate and phenol/chloroform technique [34]. Unengorged nymphal ticks were processed individually by boiling [21,35]. Tick DNA samples were tested by a TaqMan real-time PCR assay, targeting a 147 bp fragment of the rickettsial *gltA* gene [33,36]. Once the sample showed a positive result, the amplification of larger fragments of rickettsial genes was attempted by two conventional PCR assays. One used primers CS-78 and CS-323 targeting fragment 401 bp of the rickettsial *gltA* gene [33]; the second assay, with primers Rr190.70 F and Rr190.701R, targeted a fragment (≈632 bp) of the SFG rickettsial 190 kDa outer membrane protein gene (*ompA*) [37]. PCR products were DNA sequenced in an ABI automated sequencer (model ABI 3500 Genetic Analyzer; Applied Biosystems/Thermo Fisher Scientific Foster City, CA, USA) with the same primers used for PCR. The resultant sequences were submitted for BLAST analysis to infer closest identities to available sequences of *Rickettsia* spp. in GenBank.

Samples that were negative when using the real-time PCR assay were tested by conventional PCR, targeting a fragment (≈460 bp) of the tick mitochondrial 16S rRNA gene [38] with the purpose of validating the viability of the extracted DNA. If no product was amplified by this PCR assay from any of the tick DNA samples, the tick sample was discarded. In addition, we used this PCR assay to generate 16S rRNA gene partial DNA sequences to confirm the morphological identification of some tick species that were found infected by rickettsiae. 

### 2.6. Serology

Sera samples from dogs, rodents and marsupial were tested by immunofluorescence assay (IFA), using crude antigens of the following six *Rickettsia* species from Brazil: *R. rickettsii* strain Taiaçu, *R. parkeri* s.s. strain At24, *Rickettsia amblyommatis* strain Ac37, *Rickettsia rhipicephali* strain HJ5, *Rickettsia felis* strain Pedreira, and *Rickettsia bellii* strain Mogi, as described elsewhere [23]. For this purpose, each serum was initially tested at the 1:64 dilution in phosphate buffered saline (PBS). Slides were incubated with fluorescein isothiocyanate-labelled rabbit anti-dog IgG (Sigma, St Louis, MO, USA), goat anti-mouse IgG (Sigma), sheep anti-opossum IgG (CCZ, São Paulo, Brazil) and rabbit anti-guinea pig IgG (Sigma) for canine, rodent, marsupial and *Cavia aperea* sera, respectively, as previously reported [21]. Serum samples that were reactive at the 1:64 dilution were titrated in in two-fold increments to determine the endpoint IgG titers to each of the six *Rickettsia* antigens. If a serum reacted to a *Rickettsia* species with an endpoint titer that was at least 4-fold higher than the endpoint titers to the other five *Rickettsia* species, the highest titer was considered probably homologous to the first *Rickettsia* species or to a very closely related genotype [23,39]. A negative control serum (previously shown to be non-reactive) and a positive control serum (known reactive serum) were included at the 1:64 dilution in each IFA slide.

### 2.7. Correlation of Rickettsial Infection in A. tigrinum Populations

Based on the results of rickettsial detection in *A. tigrinum* ticks of the present study, we surveyed previous studies reporting *R. parkeri* and ‘*Candidatus* R. andeanae’ infections in different *A. tigrinum* populations from South America. The data were tabulated, and a Spearman test was applied to test the correlation between the infection rates of two *Rickettsia* species in *A. tigrinum*. Analyses were performed by using the R software (R Core Team, 2022, Vienna, Austria).

## 3. Results

### 3.1. Ticks from Domestic Dogs

Since the three field campaigns (June 2013, October 2013, January 2014) encompassed a study period of seven months, all data were pooled for presentation. Among a total of 70 canine examinations throughout the study, 30% (21/70) revealed tick infestations. The collected ticks were identified as adults of *Amblyomma aureolatum* (18 females and 3 males on eight dogs, 11% infestation rate), adults of *A. tigrinum* (47 females and 27 males on 10 dogs, 14% infestation), and adults of *Rhipicephalus sanguineus* s.s. (12 females and 11 males on 10 dogs, 14% infestation). 

### 3.2. Ticks from Wild Mammals and Vegetations

During the whole study period, a total of 34 small mammals of four species [one Didelphidae marsupial, *Cryptonanus chacoensis* (Chacoan gracile opossum); two Cricetidae rodents, *Akodon azarae* (Azara’s grass mouse) and *Oligoryzomys nigripes* (pygmy rice rat); and one Caviidae rodent, *Cavia aperea* (Brazilian guinea pig)] were captured, none of them showed tick infestation. This single *C. aperea* specimen was a fortuitous collection because it was removed from the mouth of a domestic dog inside the PEE during our field work. The only tick collected from a wild mammal was an adult male of *Amblyomma ovale*, collected from a *Cerdocyon thous* (crab-eating fox) that was found to be run over in front of the PEE entrance. 

From the vegetation, we collected five tick specimens that were identified as *A. aureolatum* (one nymph, two adult males) and two *A. tigrinum* (one nymph, one adult male).

### 3.3. Rickettsial Infection in Ticks

Isolation of rickettsiae in Vero cell culture was attempted individually with three adult specimens of *A. tigrinum* collected from dogs. However, no isolate was obtained.

A total of 102 tick specimens were tested by real-time PCR, resulting in the detection of rickettsial DNA in 51 samples, giving an overall infection rate of 50%. Considering each tick species and stage separately, rickettsial DNA was detected in 5% (1/20) of *A. aureolatum* adults, and in 82% (50/61) of *A. tigrinum* adults (Table 1). These ticks were further tested by conventional PCR, targeting fragments of the *gltA* and *ompA* rickettsial genes. PCR products generated reliable DNA sequences from 22 ticks. By BLAST analysis, the *gltA* (350 bp without primer sequences) and *ompA* (588 bp without primer sequences) fragments that were amplified from one adult of *A. aureolatum* and 21 adults of *A. tigrinum* were 100% identical to the available sequences of ‘*Candidatus* R. andeanae’ in GenBank (JN180849 and KF179352, respectively). Based on rickettsial confirmation by DNA sequences, the infection rate by ‘*Candidatus* R. andeanae’ in *A. tigrinum* ticks was at least 34% (21/61). The 21 ‘*Candidatus* R. andeanae’-infected ticks include the three specimens that were processed for isolation of rickettsiae in Vero cell culture.

### 3.4. Serology

A total of 70 individual serum samples (36 dogs and 34 small mammals) were tested by IFA, employing six rickettsial antigens. The proportions of reactive sera to rickettsial antigens were 7% for *R. parkeri* and *R*. *rhipicephali*, 9% for *R. rickettsii*, 6% for *R. amblyommatis* and *R. felis*, and 3% for *R. bellii* (Table 2). Among 36 dogs, five (14%) reacted to *R. parkeri* (endpoint titers: 512–8192) and *R. rickettsii* (128–8192), four (11%) to *R. amblyommatis* (64–8192), *R. rhipicephali* (256–1024) and *R. felis* (128-4096) and only two (6%) to *R. bellii* (64–128). Regarding the small mammals, only one *C. aperea* showed seroreactivity to *R. rickettsii* (endpoint titer: 128) and *R. rhipicephali* (64), whereas none of the 21 *A. azarae*, the one *C. chacoensis* and the 11 *O. nigripes* were reactive to any rickettsial antigen (Table 2).

Four dogs had endpoint titers to *R. parkeri* that were at least 4-fold higher than the endpoint titers to the other five *Rickettsia* species, indicating a possible homologous reaction to *R. parkeri* or a closely related genotype (Table 2).

### 3.5. Accession Numbers

DNA representative sequences generated in this study have been deposited in GenBank under the accession numbers KX434746–KX434747 for the 16S rRNA gene of *A. aureolatum* and *A. tigrinum*, KX434742–KX434743 for the *gltA* gene, and KX434737–KX434738 for the *ompA* gene of ‘*Candidatus* R. andeanae’. Voucher tick specimens have been deposited in the tick collection ‘Coleção Nacional de Carrapatos Danilo Gonçalves Saraiva’ (CNC), University of São Paulo, Brazil, under the accession numbers CNC 3322 and CNC 3332–3333.

### 3.6. Correlation of Rickettsial Infection in A. tigrinum Populations

With the inclusion of the present study, we tabulated 10 studies that reported rickettsial infection in *A. tigrinum* populations from South America (Table 3). In four studies, only *R. parkeri* was detected, and in four other studies, only ‘*Candidatus* R. andeanae’ was detected. Two other studies reported the presence of the two rickettsiae at disproportionate infection rates in the same *A. tigrinum* population. There was a strong negative correlation (rho: −0.76) between the ranked infection rates by *R. parkeri* and ‘*Candidatus* R. andeanae’ in populations of *A. tigrinum.* This is, when there is a high rate of infection by ‘*Candidatus* R. andeanae’, the prevalence of *R. parkeri* tends to be low or null, and vice versa.

## 4. Discussion

Through the sampling of domestic dogs, small mammals and ticks within and around a natural area of the Pampa biome in southern Brazil, we detected the following three tick species infesting the dogs: *A. aureolatum*, *A. tigrinum* and *R. sanguineus* s.s. The former two species were found to harbor the SFG agent ‘*Candidatus* R. andeanae’, revealed by DNA sequences in 34% (21/61) of the *A. tigrinum* ticks and 5% (1/20) of the *A. aureolatum* adult ticks. Surprisingly, no tick was found infesting small mammals, although this vertebrate group has been reported as hosts for immature stages of *A. aureolatum* and *A. tigrinum* [46]. This negative result could be related to low sampling, season or/and to host preference of the local populations of *A. aureolatum* and *A. tigrinum.* For instance, in an Atlantic Forest area of southeastern Brazil, immature stages of *A. aureolatum* were reported on several passerine birds, but never in the small mammals that were trapped concomitantly in the same sites [47]. In a study with *A. tigrinum* in Argentina, birds were reported as frequent hosts for both larvae and nymphs, whereas Caviidae rodents were preferred hosts for nymphs, and Cricetidae rodents for larvae [48]. Further studies with ticks in the PEE should consider the evaluation of birds and a larger sample of rodents, especially Caviidae, for a longer sampling period to gather information on host usage of *A. aureolatum* and *A. tigrinum* in a preserved region of the Pampa biome. 

Molecular analyses of ticks revealed the SFG agent ‘*Candidatus* R. andeanae’ in *A. tigrinum* ticks at a high infection rate, at least 34%. Notably, no *R. parkeri* was detected in these ticks. This result contrasts with two recent studies in the Brazilian Pampa biome, which reported absence of ‘*Candidatus* R. andeanae’, and *R. parkeri* infection rates of 28 and 46% of the *A. tigrinum* ticks [4,17]. Noteworthy, while the present study was conducted in a more preserved area of Pampa (the PEE), the previous two studies were conducted in a spotted fever-endemic area, where the Pampa has been highly degraded due to anthropic alterations (agriculture, cattle farms). The distance between this endemic area and the present study area is ≈250 Km. A rickettsial survey among *A. tigrinum* ticks from degraded areas of the Pampa biome in Uruguay also failed to detect ‘*Candidatus* R. andeanae’ and, at the same time, reported a *R. parkeri-*infection rate of 67% [14]. On the other hand, in a more preserved area of the Pampa biome in Argentina (Esteros del Iberá ecoregion), ‘*Candidatus* R. andeanae’ was detected in 100% of the *A. tigrinum* ticks [45]. These preliminary data suggest that the presence of the human pathogen *R. parkeri* under high infection rates in populations of *A. tigrinum* of the Pampa biome could be associated with environmental degradation of anthropic origin, a condition yet to be evaluated in further studies. Interestingly, previous studies in southeastern Brazil demonstrated that the endemic areas for Brazilian spotted fever (caused by *R. rickettsii*) were associated with forest degradation, which might have favored the growth of tick vector populations (either *A. aureolatum* or *Amblyomma sculptum*) and/or the encounter of the tick with vertebrate hosts that act as rickettsial amplifiers [47,49]. 

Our serological analyses showed that, at most, 14% of the dogs were exposed to SFG antigens, including *R. parkeri,* for which four dogs presented this agent as the possible antigen involved in a homologous reaction (PAIHR) (Table 2). In contrast, 100% of 18 dogs were seroreactive to *R. parkeri* (12 dogs showing *R. parkeri* as the PAIHR) in a spotted fever-endemic area of the Pampa biome, where 46% of the *A. tigrinum* ticks were infected by *R. parkeri* [17]. In another study in Brazil, where 92 (86%) out of 104 maned wolves (*C. brachyurus*) were infested by *A. tigrinum* and at least 4% of the ticks were infected by *R. parkeri,* 65 (83%) out of 78 maned wolves were seroreactive to *R. parkeri,* with 30 individuals showing *R. parkeri* as the PAIHR [42]. Thus, our canine serological results are congruent with the lack of detection of *R. parkeri* in the sampled ticks and, at the same time, suggest that *R. parkeri* might be present in the study area at very low infection rates in the tick populations. This statement might also be related to the fact that all but one of the small mammals were serologically non-reactive to SFG rickettsiae.

Through the analysis of infection rates by *R. parkeri* and ‘*Candidatus* R. andeanae’ in 10 populations of *A. tigrinum* (Table 3)*,* there was a significant negative correlation between the tick infection by these two SFG agents. While *R. parkeri* is a recognized human pathogen transmitted by ticks [50], ‘*Candidatus* R. andeanae’ is likely an endosymbiont, possibly not tick-transmitted. This statement relies on recent studies with infected *Amblyomma maculatum* ticks*,* which showed that ‘*Candidatus* R. andeanae’ was not detected in the tick salivary glands [51] and was not efficiently transmitted to the host skin during tick feeding [52]. Our results are corroborated by studies with North American populations of *A. maculatum*, in which *R. parkeri-*infection rates tended to be inversely related to the prevalence of infection by ‘*Candidatus* R. andeanae’ [50]. In fact, when >40% of the *A. maculatum* ticks were infected by ‘*Candidatus* R. andeanae’, the *R. parkeri-*infection rate was zero. On the other hand, when infection rates by ‘*Candidatus* R. andeanae’ were between 0 and <5%, the tick populations showed *R. parkeri-*infection rates ranging from 8 to 52% [13]. As proposed for *A. maculatum* [13], high infection rates by ‘*Candidatus* R. andeanae’ might promote the exclusion of *R. parkeri* from *A. tigrinum* populations. The mechanisms for such exclusion are yet to be elucidated. 

Transstadial and transovarial perpetuations are the main mechanisms for maintaining SFG rickettsiae in tick populations; however, several studies with different *Rickettsia* and tick species have reported decreasing rates of transstadial passage and/or transovarial transmission of one rickettsial agent in ticks that were previously infected by another rickettsial agent [53,54,55,56,57]. Although this interference effect has not been evaluated between ‘*Candidatus* R. andeanae’ and *R. parkeri*, it is possible that these two agents interfere with each other through transstadial and/or transovarial perpetuation in *A. tigrinum* ticks, as has been demonstrated between *Rickettsia peacockii* and *R. rickettsii* in *Dermacentor andersoni* [53]*,* between *Rickettsia montanensis* and *R. rhipicephali* in *Dermacentor variabilis* [54], between *R. amblyommatis* and *R. rickettsii* in *Amblyomma americanum* [55], between *R. amblyommatis* and *R. parkeri* in *A. americanum* [56], and between *R. bellii* and *R. rickettsii* in *Amblyomma dubitatum* [57]. Indeed, the mechanisms of such interference, related to competition between *Rickettsia* species within a tick, have not yet been elucidated. 

Although we did not test antigens of ‘*Candidatus* R. andeanae’ for serological evaluation of animals in the present study, this agent belongs to the SFG and is phylogenetically closer to *R. rhipicephali* and *R. amblyommatis* than to the remaining *Rickettsia* species tested as antigens in the present study [58]. Therefore, had the dogs and small mammals been infected by ‘*Candidatus* R. andeanae’ in the study area, it is likely that at least some of them would have presented endpoint titers higher to *R. amblyommatis* or *R. rhipicephali* than to the remaining rickettsial antigens. On the other hand, our results are in accordance with the above-mentioned studies that reported that ‘*Candidatus* R. andeanae’ is not tick-transmitted. Finally, we failed to isolate rickettsia in Vero cell cultures that were inoculated with ‘*Candidatus* R. andeanae’-infected *A. tigrinum* ticks. This result is also congruent with previous studies that reported unsuccessful isolation or limited propagation without stability of ‘*Candidatus* R. andeanae’ in mammal cell lines, including Vero [58,59,60], which reinforces the possible role of this agent as a tick endosymbiont. 

## 5. Conclusions

Despite the recent emergence of *R. parkeri* s.s.—transmitted by *A. tigrinum*—as a major agent of spotted fever illness in the South American Pampa, the present study provides epidemiological data to support that the PEE, a natural Pampa reserve, is not endemic for *R. parkeri* rickettsiosis. This statement is supported by the lack of detection of *R. parkeri-*infected ticks and the relatively low *R. parkeri* serological reactiveness of domestic dogs and small mammals in the sampled areas. Based on studies that evaluated rickettsial infection in 10 populations of *A. tigrinum* from South America, we detected a strong negative correlation between the infection rates by *R. parkeri* and the non-pathogenic agent ‘*Candidatus* R. andeanae’. Hence, our findings that at least 34% of the *A. tigrinum* ticks from the PEE were infected by ‘*Candidatus* R. andeanae’ might be the primary factor contributing to the non-endemic status of the area for *R. parkeri* rickettsiosis. This condition might be related to the preserved landscape of the PPE as a Pampa reserve, since more degraded areas of this biome have experienced high infection rates of *R. parkeri* in *A. tigrinum* ticks, which have resulted in high canine exposure to *R. parkeri* and human spotted fever illness. 

## Figures and Tables

**Table 1:** Molecular detection of rickettsial DNA in ticks collected in Barra do Quaraí municipality, state of Rio Grande do Sul, Brazil, from June 2013 to January 2014.

Tick Species	Tick Stage	Source	No. Ticks with Rickettsial DNA/No. Tested Ticks (%)	*Rickettsia* Species Identified by DNA Sequencing
*Amblyomma aureolatum*	Nymphs	Vegetation	0/1 (0)	
	Adults	Vegetation/Dogs	1/20 (5)	‘*Candidatus* R. andeanae’
*Amblyomma ovale*	Adults	*Cerdocyon thous*	0/1 (0)	
*Amblyomma tigrinum*	Adults	Vegetation/Dogs	50/61 (82) *	‘*Candidatus* R. andeanae’
*Rhipicephalus sanguineus*	Adults	Dogs	0/19 (0)	
TOTAL			51/102 (50)	

* Among the 50 PCR-positive ticks, reliable sequences of ‘*Candidatus* R. andeanae’ were generated from only 21 ticks, giving an infection rate of at least 34%.

**Table 2:** Seroreactivity to six *Rickettsia* species of animals from Barra do Quaraí municipality, state of Rio Grande do Sul, a non-endemic area for Brazilian spotted fever, from June 2013 to January 2014.

Dogs and Small Mammal Species (No. Tested Specimens)	No. Seroreactive Animals to Each *Rickettsia* Species ^a^ (% Seroreactivity)						No. Animals with PAIHR ^b^
	R.pa	R.ri	R.am	R.rh	R.fe	R.be	
Dogs (36)	5 (14)	5 (14)	4 (11)	4 (11)	4 (11)	2 (6)	4 R.pa
*Akodon azarae* (21)	0	0	0	0	0	0	
*Cavia aperea* (1)	0	1 (100)	0	1 (100)	0	0	
*Cryptonanus chacoensis* (1)	0	0	0	0	0	0	
*Oligoryzomys nigripes* (11)	0	0	0	0	0	0	
TOTAL (70)	5 (7)	6 (9)	4 (6)	5 (7)	4 (6)	2 (3)	

^a^ R.pa, *Rickettsia parkeri*; R.ri, *Rickettsia rickettsii*; R.am, *Rickettsia amblyommatis*; R.rh, *Rickettsia rhipicephali*; R.fe, *Rickettsia felis*; R.be, *Rickettsia bellii*. ^b^ PAIHR, possible antigen involved in a homologous reaction. A homologous reaction was determined when the endpoint titer to a *Rickettsia* species was at least four-fold higher than the endpoint titers observed for the other five *Rickettsia* species. In this case, the *Rickettsia* species (or a very closely related genotype) involved in the highest endpoint titer was considered the PAIHR.

**Table 3:** Infection rates by *Rickettsia parkeri* and ‘*Candidatus* Rickettsia andeanae’ reported for adult ticks of different populations of *Amblyomma tigrinum* in South America.

No. Tested Ticks	Infection Rates (%) of *A. tigrinum* Adult Ticks		Country	Reference
	*R. parkeri*	‘*Ca.* R. andeanae’		
6	67	0	Uruguay	[14]
13	46	0	Brazil	[17]
47	28	0	Brazil	[4]
81	3	0	Argentina	[40]
41	54	2 ^a^	Bolivia	[41]
63	5 *^b^*	17 ^b^	Brazil	[42]
44	0	48	Argentina	[43]
31	0	65	Chile	[44]
12	0	100	Argentina	[45]
61	0	34	Brazil	This study

^a^ The 2% value refers to a single adult specimen of *A. tigrinum* that was reported to be infected by *Rickettsia aeschlimannii*; however, because this rickettsial agent has been restricted to the Old World and is genetically close to ‘*Candidatus* R. andeane’, we are tentatively considering this report as probably the latter agent until the presence of the former agent is confirmed in South America. ^b^ Values refer to minimal infection rates, as ticks were tested in pools.

## Data Availability

The data presented in this study are available within the article.
